# Hexaaqua­cadmium(II) dipicrate monohydrate

**DOI:** 10.1107/S1600536809015049

**Published:** 2009-05-07

**Authors:** S. Natarajan, K. Moovendaran, S. A. Martin Britto Dhas, J. Suresh, P. L. Nilantha Lakshman

**Affiliations:** a Department of Physics, Madurai Kamaraj University, Madurai 625 021, India; b Department of Physics, The Madura College, Madurai 625 011, India; c Department of Food Science and Technology, Faculty of Agriculture, University of Ruhuna, Mapalana, Kamburupitiya 81100, Sri Lanka

## Abstract

In the structure of the title compound, [Cd(H_2_O)_6_](C_6_H_2_N_3_O_7_)_2_·H_2_O, the Cd^II^ ion is located on an inversion center and is coordinated by six water mol­ecules in an octa­hedral geometry. The picrate anions have no coordination inter­actions with the Cd^II^ ion. The three nitro groups are twisted away from the attached benzene ring, making dihedral angles of 17.89 (3), 27.94 (4) and 13.65 (3)°. There are numerous O—H⋯O hydrogen bonds in the crystal structure, involving coordinated and uncoordinated water molecules.

## Related literature

Picric acid forms salts with many organic and metallic cations, see: Gartland *et al.* (1974 ▶). Crystal structures have been reported for NH_4_ and K picrates (Maartmann-Moe, 1969 ▶), thallium picrate (Herbstein *et al.*, 1977 ▶), manganese picrate (Liu *et al.*, 2008 ▶) and zinc picrate (Natarajan *et al.*, 2008 ▶). For bond angles in picric acid, see: Yang *et al.* (2001 ▶).
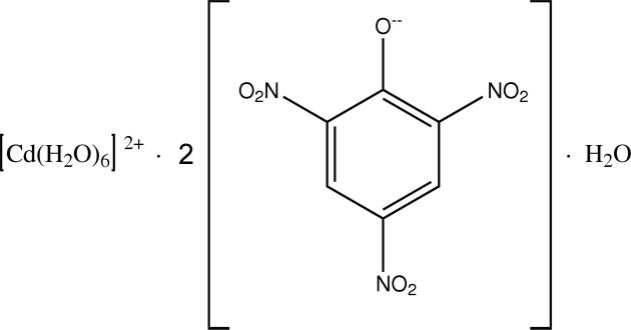


## Experimental

### 

#### Crystal data

[Cd(H_2_O)_6_](C_6_H_2_N_3_O_7_)_2_·H_2_O
*M* *_r_* = 712.75
Orthorhombic, 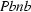

*a* = 7.2823 (2) Å
*b* = 13.2249 (4) Å
*c* = 25.3798 (8) Å
*V* = 2444.27 (13) Å^3^
*Z* = 4
Mo *K*α radiation
μ = 1.01 mm^−1^
*T* = 293 K
0.18 × 0.15 × 0.11 mm

#### Data collection

Nonius MACH-3 diffractometer
Absorption correction: ψ scan (North *et al.*, 1968 ▶) *T*_min_ = 0.834, *T*_max_ = 0.895
2247 measured reflections
2142 independent reflections
1513 reflections with *I* > 2σ(*I*)
*R*_int_ = 0.010
2 standard reflections frequency: 60 min intensity decay: none

#### Refinement

*R*[*F* ^2^ > 2σ(*F* ^2^)] = 0.027
*wR*(*F* ^2^) = 0.084
*S* = 1.16
2142 reflections
220 parameters
2 restraints
H atoms treated by a mixture of independent and constrained refinement
Δρ_max_ = 0.54 e Å^−3^
Δρ_min_ = −0.38 e Å^−3^

### 

Data collection: *CAD-4 EXPRESS* (Enraf–Nonius, 1994 ▶); cell refinement: *CAD-4 EXPRESS*; data reduction: *XCAD4* (Harms & Wocadlo, 1996 ▶); program(s) used to solve structure: *SHELXS97* (Sheldrick, 2008 ▶); program(s) used to refine structure: *SHELXL97* (Sheldrick, 2008 ▶); molecular graphics: *PLATON* (Spek, 2009 ▶); software used to prepare material for publication: *SHELXL97*.

## Supplementary Material

Crystal structure: contains datablocks global, I. DOI: 10.1107/S1600536809015049/bq2135sup1.cif

Structure factors: contains datablocks I. DOI: 10.1107/S1600536809015049/bq2135Isup2.hkl

Additional supplementary materials:  crystallographic information; 3D view; checkCIF report

## Figures and Tables

**Table 1:** Hydrogen-bond geometry (Å, °)

*D*—H⋯*A*	*D*—H	H⋯*A*	*D*⋯*A*	*D*—H⋯*A*
O11—H1*W*⋯O1^i^	0.73 (5)	2.25 (6)	2.923 (4)	155 (6)
O8—H3*W*⋯O3^ii^	0.79 (5)	2.09 (5)	2.882 (4)	172 (5)
O8—H4*W*⋯O10^iii^	0.80 (2)	1.96 (2)	2.758 (5)	171 (5)
O9—H5*W*⋯O6^iv^	0.85 (5)	2.59 (5)	2.951 (4)	107 (4)
O9—H5*W*⋯O7^iv^	0.85 (5)	2.10 (5)	2.905 (5)	159 (5)
O10—H9*W*⋯O11^iv^	0.75 (6)	2.30 (5)	2.993 (5)	154 (5)
O11—H2*W*⋯O6	0.88 (6)	2.54 (5)	2.953 (4)	109 (4)
O11—H2*W*⋯O7	0.88 (6)	2.01 (6)	2.877 (4)	166 (5)
O9—H6*W*⋯O10	0.81 (4)	1.97 (2)	2.763 (5)	167 (5)
O10—H8*W*⋯O1	0.87 (7)	2.18 (7)	2.891 (4)	140 (5)
O10—H8*W*⋯O7	0.87 (7)	2.20 (7)	2.936 (4)	142 (6)

Symmetry codes: (i) 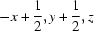; (ii) 
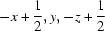
; (iii) 
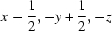
; (iv) 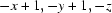.

**Fig. 1.**
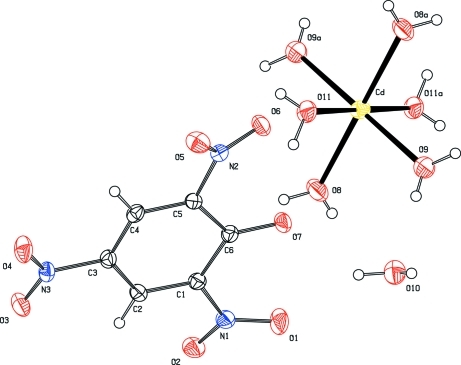
The molecular structure of the title compound, showing 30% probability displacement ellipsoids and the atom-numbering scheme.

## supplementary crystallographic information

### Comment

Picric acid forms salts with many organic and metallic cations (Gartland *et al.*, 1974). Crystal structures have been reported for isomorphous NH_4_ and K picrates (Maartmann-Moe, 1969), thallium picrate (Herbstein *et al.*, 1977), recently for manganese picrate (Liu *et al.*, 2008) and zinc picrate (Natarajan *et al.*, 2008). This work is part of a systematic investigation on the structures of the metal complexes of picric acid.

In the structure of the title compound, each Cd^II^ ion is coordinated by the O atoms of six water molecules (Fig. 1). The Cd—O distances range from 2.219 (3)Å to 2.299 (3)Å. The coordination polyhedra around the Cd^II^ ion can be described as a distorted octahedron. The picrate anion adopts a keto form with a C6—O7 bond distance of 1.250 (4)Å; the C1—C6 [1.444 (5)Å] and C5—C6 [1.454 (5)Å] bond distances are longer than the other C—C bond lengths of the benzene ring. The three nitro groups are twisted out of the attached benzene ring by 17.89 (3)° [N1/O1/O2], 27.94 (4)° [N2/O5/O6] and 13.65 (3)° [N3/O3/O4]. The twisting of these nitro groups may be attributed to the O—H···O hydrogen bonding interactions taking place between water and picrate O atoms. The C5—C6—C1 bond angle (111.8 (3)°) is smaller than the corresponding angle in picric acid (116.4 (5)°; Yang *et al.*, 2001). The packing of the molecules is governed by the large number of O—H···O hydrogen bonds (Table 1).

### Experimental

Colorless needle-shaped single crystals of the title compound were grown from a saturated aqueous solution containing picric acid and cadmium chloride in a 1:1 stoichiometric ratio.

### Refinement

O-bound H atoms were located in a difference Fourier map and their positional parameters were refined, with *U*_iso_(H) = 1.5*U*_eq_(O). C-bound H atoms were placed at calculated positions and allowed to ride on their carrier atoms, with C—H = 0.93 Å, and *U*_iso_ = 1.2*U*_eq_(C).

### Crystal data

**Table:**

[Cd(H_2_O)_6_](C_6_H_2_N_3_O_7_)_2_·H_2_O	*F*(000) = 1432
*M**_r_* = 712.75	*D*_x_ = 1.937 Mg m^−^^3^
Orthorhombic, *P**b**n**b*	Mo *K*α radiation, λ = 0.71073 Å
Hall symbol: -P 2bc 2ab	Cell parameters from 25 reflections
*a* = 7.2823 (2) Å	θ = 3–25°
*b* = 13.2249 (4) Å	µ = 1.01 mm^−^^1^
*c* = 25.3798 (8) Å	*T* = 293 K
*V* = 2444.27 (13) Å^3^	Block, colourless
*Z* = 4	0.18 × 0.15 × 0.11 mm

### Data collection

**Table:**

Nonius MACH-3 diffractometer	1513 reflections with *I* > 2σ(*I*)
Radiation source: fine-focus sealed tube	*R*_int_ = 0.010
graphite	θ_max_ = 25.0°, θ_min_ = 2.9°
ω–2θ scans	*h* = 0→8
Absorption correction: ψ scan (North *et al.*, 1968)	*k* = 0→15
*T*_min_ = 0.834, *T*_max_ = 0.895	*l* = −1→30
2247 measured reflections	2 standard reflections every 60 min
2142 independent reflections	intensity decay: none

### Refinement

**Table:**

Refinement on *F*^2^	Secondary atom site location: difference Fourier map
Least-squares matrix: full	Hydrogen site location: inferred from neighbouring sites
*R*[*F*^2^ > 2σ(*F*^2^)] = 0.027	H atoms treated by a mixture of independent and constrained refinement
*wR*(*F*^2^) = 0.084	*w* = 1/[σ^2^(*F*_o_^2^) + (0.0314*P*)^2^ + 3.7212*P*] where *P* = (*F*_o_^2^ + 2*F*_c_^2^)/3
*S* = 1.16	(Δ/σ)_max_ < 0.001
2142 reflections	Δρ_max_ = 0.54 e Å^−^^3^
220 parameters	Δρ_min_ = −0.38 e Å^−^^3^
2 restraints	Extinction correction: *SHELXL97* (Sheldrick, 2008), Fc^*^=kFc[1+0.001xFc^2^λ^3^/sin(2θ)]^-1/4^
Primary atom site location: structure-invariant direct methods	Extinction coefficient: 0.0029 (2)

### Special details

**Table:**

Geometry. All e.s.d.'s (except the e.s.d. in the dihedral angle between two l.s. planes) are estimated using the full covariance matrix. The cell e.s.d.'s are taken into account individually in the estimation of e.s.d.'s in distances, angles and torsion angles; correlations between e.s.d.'s in cell parameters are only used when they are defined by crystal symmetry. An approximate (isotropic) treatment of cell e.s.d.'s is used for estimating e.s.d.'s involving l.s. planes.
Refinement. Refinement of *F*^2^ against ALL reflections. The weighted *R*-factor *wR* and goodness of fit *S* are based on *F*^2^, conventional *R*-factors *R* are based on *F*, with *F* set to zero for negative *F*^2^. The threshold expression of *F*^2^ > σ(*F*^2^) is used only for calculating *R*-factors(gt) *etc*. and is not relevant to the choice of reflections for refinement. *R*-factors based on *F*^2^ are statistically about twice as large as those based on *F*, and *R*- factors based on ALL data will be even larger.

### Fractional atomic coordinates and isotropic or equivalent isotropic displacement parameters (Å^2^)

**Table:**

	*x*	*y*	*z*	*U*_iso_*/*U*_eq_	
Cd	0.0000	0.5000	0.0000	0.03236 (15)	
O11	0.1978 (4)	0.5718 (3)	0.05990 (11)	0.0393 (7)	
O1	0.4465 (5)	0.2509 (2)	0.11321 (10)	0.0581 (9)	
O5	0.6784 (4)	0.6893 (2)	0.18800 (10)	0.0430 (7)	
O7	0.4862 (4)	0.4516 (2)	0.10554 (9)	0.0390 (7)	
O2	0.3391 (4)	0.1969 (2)	0.18648 (10)	0.0435 (7)	
O6	0.5121 (4)	0.6525 (2)	0.12019 (9)	0.0415 (7)	
O4	0.5772 (5)	0.5100 (2)	0.34801 (10)	0.0463 (7)	
N3	0.5092 (4)	0.4393 (2)	0.32448 (11)	0.0297 (7)	
O8	−0.0269 (6)	0.3512 (2)	0.04266 (13)	0.0609 (10)	
O9	0.2500 (5)	0.4420 (3)	−0.03995 (13)	0.0554 (9)	
O3	0.4428 (4)	0.3650 (2)	0.34660 (10)	0.0430 (7)	
O10	0.5209 (5)	0.3302 (2)	0.00923 (13)	0.0457 (8)	
N2	0.5770 (4)	0.6320 (2)	0.16356 (11)	0.0301 (7)	
C4	0.5398 (5)	0.5320 (3)	0.24176 (14)	0.0280 (8)	
H4	0.5694	0.5898	0.2608	0.034*	
N1	0.4123 (5)	0.2622 (2)	0.15992 (11)	0.0336 (7)	
C2	0.4606 (5)	0.3554 (3)	0.23989 (13)	0.0290 (8)	
H2	0.4336	0.2958	0.2577	0.035*	
C3	0.5024 (5)	0.4423 (3)	0.26750 (12)	0.0279 (8)	
C6	0.4911 (5)	0.4481 (3)	0.15474 (13)	0.0272 (7)	
C1	0.4592 (5)	0.3581 (3)	0.18566 (13)	0.0286 (8)	
C5	0.5328 (5)	0.5350 (3)	0.18776 (13)	0.0258 (8)	
H8W	0.517 (8)	0.337 (5)	0.043 (3)	0.09 (2)*	
H1W	0.172 (8)	0.607 (4)	0.081 (2)	0.07 (2)*	
H3W	−0.009 (7)	0.350 (4)	0.074 (2)	0.065 (16)*	
H5W	0.302 (7)	0.475 (4)	−0.0643 (19)	0.063 (16)*	
H2W	0.276 (8)	0.536 (4)	0.0788 (19)	0.067 (17)*	
H6W	0.317 (5)	0.402 (3)	−0.0255 (15)	0.045 (13)*	
H9W	0.612 (8)	0.349 (4)	−0.0004 (19)	0.06 (2)*	
H4W	−0.007 (7)	0.297 (2)	0.0302 (19)	0.065 (17)*	

### Atomic displacement parameters (Å^2^)

**Table:**

	*U*^11^	*U*^22^	*U*^33^	*U*^12^	*U*^13^	*U*^23^
Cd	0.0329 (2)	0.0278 (2)	0.0363 (2)	0.00038 (17)	−0.00396 (18)	−0.00312 (16)
O11	0.0433 (17)	0.0432 (17)	0.0314 (14)	0.0014 (14)	−0.0075 (13)	−0.0079 (14)
O1	0.109 (3)	0.0353 (16)	0.0295 (15)	−0.0106 (17)	0.0057 (16)	−0.0099 (12)
O5	0.0509 (18)	0.0310 (15)	0.0470 (15)	−0.0088 (13)	−0.0122 (14)	0.0002 (13)
O7	0.0649 (19)	0.0298 (13)	0.0221 (12)	0.0003 (13)	−0.0042 (13)	−0.0018 (11)
O2	0.0614 (19)	0.0269 (15)	0.0423 (15)	−0.0055 (14)	−0.0051 (14)	0.0004 (12)
O6	0.066 (2)	0.0324 (14)	0.0260 (13)	−0.0018 (14)	−0.0045 (13)	0.0039 (11)
O4	0.0609 (18)	0.0510 (18)	0.0270 (13)	−0.0151 (16)	−0.0020 (13)	−0.0087 (13)
N3	0.0297 (16)	0.0375 (18)	0.0220 (14)	−0.0003 (15)	−0.0011 (14)	−0.0031 (13)
O8	0.118 (3)	0.0330 (16)	0.0312 (16)	0.004 (2)	−0.0078 (19)	0.0040 (13)
O9	0.058 (2)	0.063 (2)	0.0451 (17)	0.0244 (19)	0.0174 (17)	0.0129 (17)
O3	0.0582 (18)	0.0428 (16)	0.0281 (13)	−0.0079 (15)	−0.0016 (13)	0.0068 (12)
O10	0.051 (2)	0.0435 (17)	0.0426 (19)	−0.0074 (16)	0.0016 (16)	−0.0024 (14)
N2	0.0346 (16)	0.0271 (16)	0.0286 (15)	0.0041 (14)	0.0019 (14)	−0.0006 (13)
C4	0.026 (2)	0.0300 (17)	0.0278 (18)	0.0014 (15)	−0.0025 (14)	−0.0070 (15)
N1	0.0434 (19)	0.0268 (16)	0.0306 (16)	0.0010 (15)	−0.0037 (15)	−0.0016 (14)
C2	0.033 (2)	0.0288 (17)	0.0255 (18)	0.0020 (16)	−0.0023 (14)	0.0026 (14)
C3	0.0279 (17)	0.0334 (19)	0.0225 (16)	0.0027 (16)	−0.0020 (16)	−0.0017 (14)
C6	0.0287 (18)	0.0272 (17)	0.0256 (17)	0.0017 (15)	−0.0010 (16)	−0.0022 (14)
C1	0.035 (2)	0.0238 (17)	0.0268 (17)	0.0012 (16)	−0.0043 (15)	−0.0030 (14)
C5	0.028 (2)	0.0239 (16)	0.0256 (16)	0.0022 (14)	−0.0013 (14)	0.0004 (14)

### Geometric parameters (Å, °)

**Table:**

Cd—O9^i^	2.221 (3)	O8—H3W	0.79 (5)
Cd—O9	2.221 (3)	O8—H4W	0.80 (2)
Cd—O8^i^	2.255 (3)	O9—H5W	0.85 (5)
Cd—O8	2.255 (3)	O9—H6W	0.81 (4)
Cd—O11	2.299 (3)	O10—H8W	0.87 (7)
Cd—O11^i^	2.299 (3)	O10—H9W	0.75 (6)
O11—H1W	0.73 (5)	N2—C5	1.459 (5)
O11—H2W	0.88 (6)	C4—C5	1.372 (5)
O1—N1	1.220 (4)	C4—C3	1.382 (5)
O5—N2	1.226 (4)	C4—H4	0.9300
O7—C6	1.250 (4)	N1—C1	1.467 (4)
O2—N1	1.219 (4)	C2—C1	1.377 (5)
O6—N2	1.228 (4)	C2—C3	1.380 (5)
O4—N3	1.216 (4)	C2—H2	0.9300
N3—O3	1.231 (4)	C6—C1	1.444 (5)
N3—C3	1.447 (4)	C6—C5	1.454 (5)
			
O9^i^—Cd—O9	180.00 (15)	H5W—O9—H6W	113 (5)
O9^i^—Cd—O8^i^	89.37 (14)	H8W—O10—H9W	108 (5)
O9—Cd—O8^i^	90.63 (14)	O5—N2—O6	123.3 (3)
O9^i^—Cd—O8	90.63 (14)	O5—N2—C5	117.6 (3)
O9—Cd—O8	89.37 (14)	O6—N2—C5	119.1 (3)
O8^i^—Cd—O8	180.00 (16)	C5—C4—C3	119.3 (3)
O9^i^—Cd—O11	93.97 (13)	C5—C4—H4	120.4
O9—Cd—O11	86.03 (13)	C3—C4—H4	120.4
O8^i^—Cd—O11	84.41 (13)	O2—N1—O1	122.7 (3)
O8—Cd—O11	95.59 (13)	O2—N1—C1	117.9 (3)
O9^i^—Cd—O11^i^	86.03 (13)	O1—N1—C1	119.4 (3)
O9—Cd—O11^i^	93.97 (13)	C1—C2—C3	119.2 (3)
O8^i^—Cd—O11^i^	95.59 (13)	C1—C2—H2	120.4
O8—Cd—O11^i^	84.41 (13)	C3—C2—H2	120.4
O11—Cd—O11^i^	180.00 (11)	C2—C3—C4	121.2 (3)
Cd—O11—H1W	126 (5)	C2—C3—N3	119.5 (3)
Cd—O11—H2W	123 (3)	C4—C3—N3	119.3 (3)
H1W—O11—H2W	96 (5)	O7—C6—C1	124.7 (3)
O4—N3—O3	123.4 (3)	O7—C6—C5	123.5 (3)
O4—N3—C3	118.9 (3)	C1—C6—C5	111.8 (3)
O3—N3—C3	117.7 (3)	C2—C1—C6	124.3 (3)
Cd—O8—H3W	118 (4)	C2—C1—N1	115.1 (3)
Cd—O8—H4W	126 (4)	C6—C1—N1	120.5 (3)
H3W—O8—H4W	110 (5)	C4—C5—C6	124.1 (3)
Cd—O9—H5W	122 (3)	C4—C5—N2	115.9 (3)
Cd—O9—H6W	121 (3)	C6—C5—N2	119.9 (3)
			
C1—C2—C3—C4	1.1 (6)	O2—N1—C1—C2	16.5 (5)
C1—C2—C3—N3	−178.2 (3)	O1—N1—C1—C2	−163.9 (4)
C5—C4—C3—C2	0.8 (6)	O2—N1—C1—C6	−160.4 (3)
C5—C4—C3—N3	−179.8 (3)	O1—N1—C1—C6	19.2 (5)
O4—N3—C3—C2	167.0 (3)	C3—C4—C5—C6	−1.1 (5)
O3—N3—C3—C2	−14.0 (5)	C3—C4—C5—N2	−179.0 (3)
O4—N3—C3—C4	−12.3 (5)	O7—C6—C5—C4	−179.6 (4)
O3—N3—C3—C4	166.7 (3)	C1—C6—C5—C4	−0.4 (5)
C3—C2—C1—C6	−2.9 (6)	O7—C6—C5—N2	−1.8 (5)
C3—C2—C1—N1	−179.7 (3)	C1—C6—C5—N2	177.4 (3)
O7—C6—C1—C2	−178.4 (4)	O5—N2—C5—C4	26.6 (5)
C5—C6—C1—C2	2.5 (5)	O6—N2—C5—C4	−153.5 (3)
O7—C6—C1—N1	−1.8 (6)	O5—N2—C5—C6	−151.4 (3)
C5—C6—C1—N1	179.1 (3)	O6—N2—C5—C6	28.5 (5)

Symmetry codes: (i) −*x*, −*y*+1, −*z*.

### Hydrogen-bond geometry (Å, °)

**Table:**

*D*—H···*A*	*D*—H	H···*A*	*D*···*A*	*D*—H···*A*
O11—H1W···O1^ii^	0.73 (5)	2.25 (6)	2.923 (4)	155 (6)
O8—H3W···O3^iii^	0.79 (5)	2.09 (5)	2.882 (4)	172 (5)
O8—H4W···O10^iv^	0.80 (2)	1.96 (2)	2.758 (5)	171 (5)
O9—H5W···O6^v^	0.85 (5)	2.59 (5)	2.951 (4)	107 (4)
O9—H5W···O7^v^	0.85 (5)	2.10 (5)	2.905 (5)	159 (5)
O10—H9W···O11^v^	0.75 (6)	2.30 (5)	2.993 (5)	154 (5)
C4—H4···O2^vi^	0.93	2.57	3.195 (4)	125
O11—H2W···O6	0.88 (6)	2.54 (5)	2.953 (4)	109 (4)
O11—H2W···O7	0.88 (6)	2.01 (6)	2.877 (4)	166 (5)
O9—H6W···O10	0.81 (4)	1.97 (2)	2.763 (5)	167 (5)
O10—H8W···O1	0.87 (7)	2.18 (7)	2.891 (4)	140 (5)
O10—H8W···O7	0.87 (7)	2.20 (7)	2.936 (4)	142 (6)

Symmetry codes: (ii) −*x*+1/2, *y*+1/2, *z*; (iii) −*x*+1/2, *y*, −*z*+1/2; (iv) *x*−1/2, −*y*+1/2, −*z*; (v) −*x*+1, −*y*+1, −*z*; (vi) *x*, *y*+1/2, −*z*+1/2.
